# Social participation in the health technology incorporation process into Unified Health System

**DOI:** 10.11606/S1518-8787.2019053001420

**Published:** 2019-12-16

**Authors:** Aline Silveira Silva, Maria Sharmila Alina de Sousa, Emília Vitória da Silva, Dayani Galato

**Affiliations:** I Universidade de Brasília. Faculdade de Ceilândia. Programa de Pós-Graduação em Ciências e Tecnologias da Saúde. Ceilândia, DF, Brasil; II Ministério da Saúde. Secretaria de Ciência, Tecnologia e Insumos Estratégicos – SCTIE. Departamento de Gestão e Incorporação de Tecnologias e Inovação em Saúde – DGITIS. Brasília, DF, Brasil; III Escola Fiocruz de Governo. Brasília, DF, Brasil; IV Universidade de Brasília. Faculdade de Ceilândia. Curso de Farmácia. Grupo de Pesquisa em Acesso aos Medicamentos e Uso Responsável – AMUR. Ceilândia, DF, Brasil

**Keywords:** Technology Assessment, Biomedical Community Participation, Unified Health System

## Abstract

**OBJECTIVE:**

To describe the current process of social participation in the incorporation of health technologies in Brazil, within the context of the Unified Health System (SUS).

**METHODS:**

A descriptive study was conducted based on the analysis of official records of the actions of the National Committee for Health Technology Incorporation into Unified Health System and its website, from the beginning of its activities in January 2012 until December 2017.

**RESULTS:**

The findings indicate that, in Brazil, there are legal instruments related to social participation in health, including the health technology assessment (HTA) field. However, its implementation is relatively recent and has been carried out gradually. In addition to the legal instruments (National Health Council representative, public consultation and public hearing forecast), other information and transparency strategies have been shown to be allied to social participation in the incorporation of health technologies. However, activities such as legally provided public hearings have not yet been carried out.

**CONCLUSIONS:**

Several actions to foster social participation were developed over the analyzed period, but they need to be evaluated in order to maintain or improve them. In addition, there is a need for more qualified social participation in the various existing spaces, including those prescribed by law.

## INTRODUCTION

Health technology assessment (HTA) is a multidisciplinary field of public policy analysis that studies the clinical, social, ethical, and economic implications of health technology development, diffusion, and use, considering aspects such as efficiency, effectiveness, safety, costs, cost-effectiveness, among others^1 , 2^. However, the ethical, legal, and social impacts directed to the social perspective are often disregarded due to clinical and economic findings^3^.

Members of the Health Technology Assessment International Global Policy Forum reported that practitioners in this area had been focusing on reporting and internal processes for a few years, interacting in a limited way with society; nowadays, however, this perspective has changed. HTA practitioners are increasingly seeking colloquial evidence, a term that covers different types of informal opinion^4^, to complement data from randomized controlled trials. The goal is to focus on various aspects, not just clinical ones, and thus increasingly adapt HTA processes to the real contexts where these technologies are or will be used^5^.

Literature proposes different terms to refer to the various groups of HTA end users, such as patients, consumers, citizens and the public. The model developed by Gauvin et al.^6^ grouped these users into two categories. The first category audience provides a social or lay perspective on health technologies that includes citizens, groups of citizens or elected representatives. The second category comprises those directly affected by a particular health condition or technology and includes patients, service users, and entities that represent them.

The model described above emphasizes three main aspects: domains of involvement (organizational policy and research), type of audience (lay and directly affected) and level of involvement (information, consultation and participation)^7^.

As patient engagement increases, individuals become more aware of their rights and begin participating in their own health care, which calls for effective means to involve civil society in decision-making on technology incorporation. Many HTA agencies in different countries have considered various ways to incorporate patients’ perspectives into their models and methods of social engagement, thus requiring a patient-centered HTA^7 , 8^.

Terminological consensus on this involvement is still lacking. International studies addressing the topic often use the terms “public and patient engagement”, “public engagement” or “patient engagement”^4^. In this study, the term “social participation” was adopted, as this is how Brazilian legislation refers to the theme, including when dealing with HTA^9^.

In Brazil, social participation is one of the guidelines of the Unified Health System (SUS) present in the Federal Constitution^10^ and is one of the principles described in Laws 8.080/1990^11^ and 8.142/1990^12^. Society built social control in SUS, with health councils and conferences, defining spaces for social participation in the formulation and deliberation of public health policy^13^. However, this participation in contemporary democratic societies is a recent fact^14^. Although community participation is constitutionally assured, the Brazilian examples of democratic health experience in this area are still punctual^15 , 16^. Moreover, according to Delduque and Bardal (2008)^15^, the social participation mentioned is not restricted to the social control of health. That is, it does not only refer to the formation of councils or health conferences, but rather to a broader participation, one inherent in the full fulfillment of citizenship, allowing citizens to truly participate, in the Habermasian sense^17 , 18^, in communicative action and healthcare decision-making processes^15^.

Law 12.401/2011 defines that the incorporation, exclusion or alteration into SUS of new drugs, products and procedures, as well as the constitution or alteration of clinical protocols and therapeutic guidelines (PCDT), are attributions of the Ministry of Health, advised by the National Committee for Health Technology Incorporation into Unified Health System (Conitec)^19^. Conitec’s operating structure is composed of two forums: plenary and executive secretary. One of the duties of the executive secretariat, performed by the Department of Management and Incorporation of Health Technologies and Inovation (DGITIS), is to promote actions that favor and stimulate social participation in the health technology incorporation into SUS^9^; this attribution dialogues with the National Policy of Social Participation and the National System of Social Participation^20^.

The same law^19^ also formalized social participation in the technology incorporation process, which previously did not have this legal provision, and there were few attempts to involve the public and the patient before Conitec^16^. This participation, therefore, currently occurs through the *Conselho Nacional de Saúde* (CNS – National Health Council), which represents SUS users, as a member of the Conitec plenary; holding a public consultation (PC) for all recommendations issued; and public hearing before the final decision is taken, in cases in which the secretary of the Ministry of Health’s Secretariat of Science, Technology and Strategic Inputs determines that the relevance of the matter justifies its realization.

However, recently, Decree 9.759/2019 was published, which “extinguishes and establishes guidelines, rules and limitations for federal public administration colleges”, an action that goes against social advances, causing insecurity regarding the maintenance of social participation in various scenarios in our country.

This study aims to describe the current state of social participation in health technology incorporation processes developed by Conitec.

## METHODS

This investigation is characterized as a case study on social participation in the technology incorporation process developed by Conitec in the context of SUS. To this end, documentary analysis^21^ and description of the actions that promoted social participation in the health technology assessment and incorporation between January 2012 and December 2017 were made. We described all actions developed by Conitec that aimed to reach society and encourage its participation, then analyzed their results. They were shown in a timeline, describing the moment of their publication, execution or implementation, in case of continuous actions.

To describe the actions taken, the number of reports to society published by Conitec, the number of public consultations performed, as well as the count and classification of the authors of the suggestions received were computed. The classification of these authors’ suggestions was performed according to the model by Gauvin et al. (2010)^6^, indicating those directly affected by a particular health condition or technology, and according to the registration of the SUS form (FormSUS).

To obtain data related to the results of Conitec’s actions, the spreadsheets and DGITIS management information system were consulted, as well as the committee’s electronic portal (www.conitec.gov.br).

This study is part of the research project entitled *O envolvimento do público e do paciente no processo de incorporação de tecnologias em saúde no Brasil* (The patient and public involvement in the Brazilian health technology incorporation process), which was approved by the Research Ethics Committee of the Faculdade de Ceilândia, University of Brasilia, under the opinion no. 2.225.660.

## RESULTS

**Figure 1:**
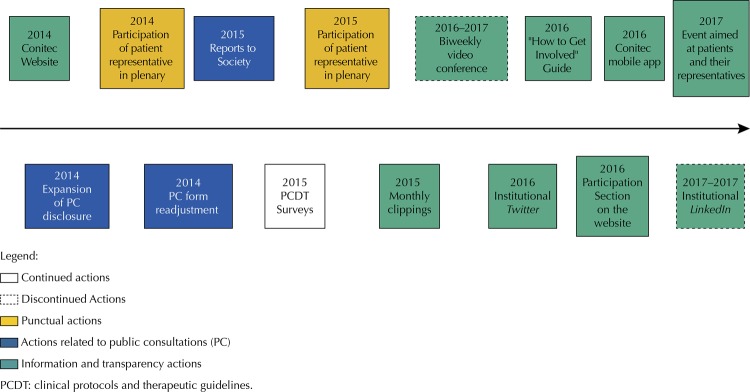
Timeline of actions involving public and patients in the evaluation and incorporation processes of technologies in SUS developed since the creation of Conitec until December 2017. Source: Conitec, 2018.

Figure 1 shows a timeline with events related to social participation in the health technology incorporation process into SUS. The actions are described in detail according to the following classification: public consultation (PC); representation of patients in plenary; surveys; and information and transparency.

### Public Consultation

According to the National Policy for Social Participation, public consultation is a “participatory mechanism, to be held within a defined period, of an advisory nature, open to any interested party, which aims to receive written suggestions from civil society on a given subject, in the form defined in its act of calling”^20^.

In the health technology incorporation into SUS, the objective of PC is to broaden the discussion on the subjects under analysis, bypassing the technical, scientific, economic and logistic aspects already identified. Also, the vision and experiences of patients, health professionals, citizens and other social actors are added to the discussion. Thus, after preparing a technical report, Conitec makes it available on its website to receive suggestions from society for 20 days. All suggestions are compiled and taken to the plenary for consideration prior to issuing the final recommendation on incorporation^22^.

In order to improve the suggestions of PC, in 2014 a new form was created to differentiate the reports of experiences and perspectives of patients, caregivers and health professionals from those technical and scientific reports.

In 2015, the DGITIS started producing reports to society, which are summarized versions of Conitec’s technical reports, made available to them at PC moment. They are designed in simplified language to improve users’ understanding of the technologies being analyzed and to encourage their participation in the HTA process. Until December 2017, 76 reports have been published to society.

**Figure 2:**
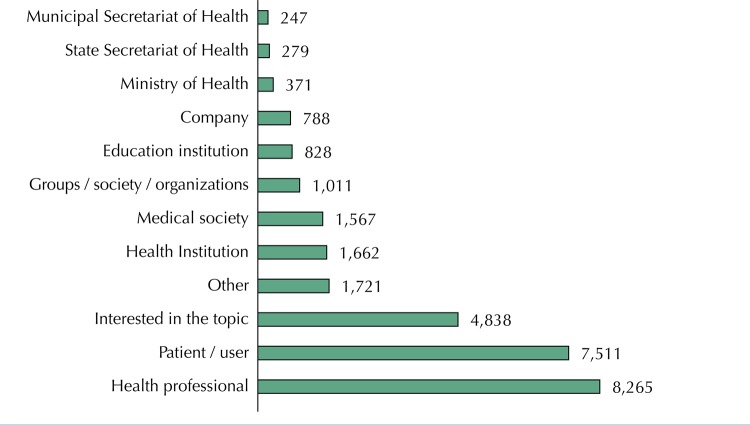
Type of public participating in public consultations conducted by the National Committee for Health Technology Incorporation (as self-declared in the public consultation forms), from 2012 to 2017. Source: Conitec, 2018.

In the period investigated, 257 PC were performed, with 42,630 suggestions by the various stakeholders, mainly family members, friends or caregivers of patients; health professionals; SUS patients/users; and actors interested in the theme ( Figure 2 ). In the authors’ analysis of the suggestions according to the classification by Gauvin et al.^6^, it is observed that almost half of the participants (44.6%, n = 19,006) fit into the category of those directly affected by a particular condition or health technology (SUS patients and users; family, friends or caregivers; and patient groups, organizations or associations).

**Figure 3:**
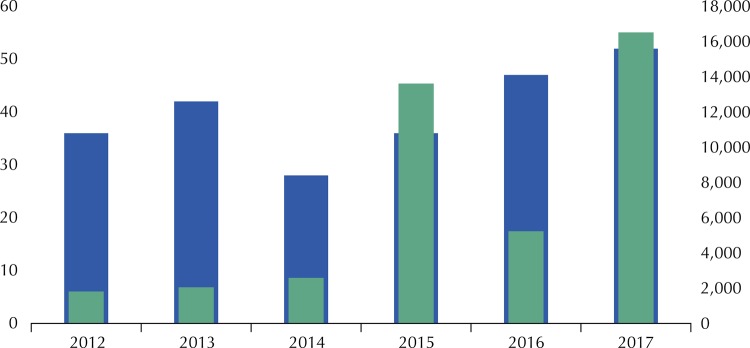
Number of public consultations and suggestions received per year until December 2017. Source: Conitec, 2018.

As of 2014 ( Figure 1 ), Conitec’s executive secretary aimed to expand PC information through some digital social media such as institutional *Twitter* , partner’s channels, institutional *LinkedIn* (which was created and closed in 2017), website and email lists. Following the implementation of these strategies, social participation in PC increased from 2,584 suggestions in 2014 to 13,619 suggestions in 2015 and 16,514 suggestions in 2017 ( Figure 3 ). In addition to the information strategy adopted, it was observed that topics of great popular appeal also interfered with the increase of consultations, such as: “Exclusion of beta interferon for the treatment of multiple sclerosis” (4,846 suggestions) and “Guidelines for pregnant women: the Caesarean section operation” (3,706 suggestions) in 2015; and “PCDT for HIV Pre-Exposure Prophylaxis (PrEP)” (3,773 suggestions) and “Dimethyl fumarate for multiple sclerosis” (1,813 suggestions) in 2017.

In some cases, the considerations received during the PC influenced the decision-making of the committee, changing the initial recommendation of non-incorporation to a final recommendation of incorporation, either by providing new scientific evidence and pricing proposals, or by bringing needs and preferences of patients who had not been included in the previously defined outcomes ( Table 1 ). However, so far, no information on the quality or content analysis of suggestions has been published, which could guide the qualification of this participation tool.

**Table 1:** Topics that had the recommendation “non-incorporation” changed to “incorporation” after analyzing the suggestions received at public consultations until December 2017.

Year	Technology	Indication	Reason for change after PC
2013	Erlotinib and gefitinib	Advanced or metastatic non-small cell lung cancer EGFR mutation	Presentation of new evidence in PC showed benefit of technology (increased progression-free survival)
2015	Subcutaneous abatacept	Moderate to severe rheumatoid arthritis	Presentation of new price proposal received at PC equated the analyzed TS with the comparator, due to its non-inferiority
2015	Rivastigmine transdermal patch	Mild and moderately severe Alzheimer’s dementia	Presentation of new price proposal received at PC equated the analyzed TS with the comparator, due to its non-inferiority
2017	Fingolimod	Recurrent remitting multiple sclerosis after beta-interferon or glatiramer therapy failure	Review of currently recommended SUS lines of treatment and availability of a generic drug
2017	Rapid-acting analog insulin	Type I *diabetes mellitus*	Reconsideration of the importance of other outcomes, incorporation recommendation made through price negotiation with the manufacturer
2017	Dimethyl Fumarate	Recurrent remitting multiple sclerosis after beta-interferon or glatiramer therapy failure	Presentation of new price proposal received at PC equated the analyzed TS with the comparator, due to its non-inferiority

### Conitec Plenary Patient Representation

The Conitec plenary has as its member a representative of the CNS. However, the participation of other representatives of society and patients may still occur in other ways, which, although scarce, are important from the perspective of the quality of suggestions — colloquial evidence — to support decision-making in plenary.

At times, when requested by the plenary, patient representatives and experts on the subject were invited to attend the plenary sessions. This participation provided Conitec with very informative colloquial evidence on the experience of patients and experts on the topic^4 , 23 , 24^.

It is important to point out that, at other times, in plenary meetings, the presence of representatives of society (professionals or patients) was accepted when requested by them. In these cases, those attendents could only follow the technical presentation of the topic, but not the subsequent discussion of the plenary. However, the law provides for a public hearing to be held when the relevance of the matter justifies it^19^. According to the National Policy for Social Participation, the public hearing is a “participatory, face-to-face, consultative mechanism open to anyone interested, with the possibility of oral expression by participants, whose purpose is to subsidize governmental decisions”^20^. However, in the period investigated, no record of use of this mechanism was found.

### Surveys

In 2015, aiming to investigate the needs and preferences of SUS users and to continue the process of improving social participation and transparency of Conitec processes, DGITIS announced the holding of surveys related to PCDT. They are available on the Conitec website, usually for 20 days, as prior consultation, still in the first stage of PCDT construction. Thus, the executive secretary can discuss and improve the initial proposal of the document by identifying aspects that may not have been previously considered. This was the way the commission itself found to implement other forms of social participation since the beginning of the PCDT elaboration process, and not only in PC.

Thus, any society actors can suggest information on certain aspects of the illnesses with which they have experience (personal or professional), improvements in health care and appeal for new technologies that should be addressed. As this participation occurs before the document is prepared, society contributes to the construction of the scope of the protocol in question. During the analyzed period, 17 surveys were conducted on the most diverse topics ( Table 2 ).

**Table 2:** Surveys related to clinical protocols and therapeutic guidelines (PCDT) conducted by Conitec until December 2017.

Survey no.	Year	Topic	No. of suggestions
01	2015	Rare disease PCDT	1,140
02	2015	Proposed scope of diagnostic and therapeutic guidelines for pesticide poisoning	38
03	2016	Proposed update of PCDT published in 2012 and 2013	1,054
04	2016	Proposed elaboration of PCDT for the care of individuals with Chagas disease	37
05	2016	PCDT scope proposal for visceral and cutaneous leishmaniasis	11
06	2016	Scope proposal for Brazilian guideline for the use of coronary stent angioplasty	10
07	2016	PCDT 2014 Update	304
08	2017	PCDT scope proposal for Rocky Mountain spotted fever	5
09	2017	PCDT scope proposal for malaria	1
10	2017	PCDT scope proposal for chikungunya	4
11	2017	Proposed scope of PCDT review and update for nicotine addiction	192
12	2017	Scope proposal of diagnostic guidelines for malignant pleura mesothelioma	12
13	2017	Preparation of Conitec technology incorporation proposal submission guide	62
14	2017	Proposed PCDT scope for hormone therapy in transsexualising process	78
15	2017	Scope proposal of the Primary Healthcare protocol – chronic pain	116
16	2017	Scope proposal of the Brazilian directive for thoracic aortic endoprostheses	0
17	2017	Scope proposal for pulmonary arterial hypertension guideline	627

### Information and Transparency

The strategies adopted to promote information and transparency are shown in Figure 1 .

The Conitec website, created at the end of 2014, was elected by its executive secretary as the basis for information and transparency initiatives^24^, as it allows access to all technical reports and suggestions received in the PC already made, as well as agendas and minutes of the plenary meetings. According to the institutional access analysis using *Google Analytics* ®, from January 2015 to December 2017, the Conitec portal had 482,524 sessions.

In the social participation section of this website, created in June 2016, it is possible to access, for example, the ongoing and closed PC and surveys, the reports to society and monthly clippings about Conitec’s activities. According to the institutional access analysis using *Google Analytics* ®, this section gained 2,306 views (until December 2017).

In 2016, Conitec launched the guide *Understanding the incorporation of health technologies into SUS: how to get involved* ^25^ available from the Social Participation section of the website, to inform and facilitate the engagement of society. The document was also printed and distributed at events, and was sent to various institutions.

In the second half of 2017, the forum “Understanding the incorporation of health technologies” took place. This event was directed to patient representatives, who had the opportunity to discuss the Conitec’s social participation process with the executive secretary’s members. A total of 103 patient representatives from associations of various diseases and regions of the country were present, who were able to better understand the points foreseen for social participation and provide suggestions for improvement, which were presented to the Conitec plenary later. All material related to the event was made available on the social participation section of the Conitec website.

In addition, between August 2016 and December 2017, DGITIS held video conferences every two weeks through a program called “Conitec in evidence”, which aimed to discuss and deepen relevant topics of technology management and evaluation with the most various actors in the area, as well as disseminating the content produced by Conitec and the *Núcleos de Avaliação de Tecnologias em Saúde* (NATS – Health Technology Assessment Centers). In addition to the participation of various institutions via videoconference, any citizen could follow the transmission of content in real time and ask questions via e-mail and institutional *Twitter* . Although the program ended in 2017, its content remains available on the Conitec website. In the analyzed period, 25 programs were carried out on various issues related to HTA, one of them being “ *Participação social para o fortalecimento da ATS: avanços e desafios* ” (Social Participation for the Strengthening of HTA: Advances and Challenges).

## DISCUSSION

There are numerous studies reporting experiences of participation and increased attention to patient involvement in HTA around the world^3 , 5 , 8^.

Engagement processes differ among HTA agencies around the world, especially in terms of domains and types of engagement, audience types, and how much that involvement influences decisions about incorporating technologies into health systems^26^.

Early Brazilian experience shows that it can adapt to the context and particularities of the national HTA. However, despite the advances already made with the formalization of social participation in HTA^8 , 19 , 22 , 27^, engaging society in these activities remains challenging.

In addition to the legally foreseen participation mechanisms, especially those related to the PC, we have observed the experimentation of various strategies by the Conitec executive secretary since their implementation.

Given that the representation of the CNS as a member of the committee is insufficient due to the impossibility of representing the public involved in all evaluated topics, and even though attempts are made to represent patients at Conitec plenary sessions when requested by the plenary, we suggest that this mechanism of (active) participation during plenary sessions, with appropriate and previously defined methodology, be strengthened. Another strategy to explore would be the public hearing, a planned mechanism^19^, but not yet used. Widely mentioned in Brazilian law as one of the main mechanisms for social participation in public management, it is consultative and allows society to express its wishes and opinions, providing subsidies for decision making^28^.

The implementation of strategies for participation, transparency, knowledge translation and social appropriation of knowledge developed by Conitec seems to stimulate the public to participate in the process of health technology incorporation. To corroborate this statement, there was an increase of more than 400% in the number of annual suggestions in PC from 2014 to 2015. Some communication actions were discontinued in 2017, such as *LinkedIn* and the “Conitec in evidence” videoconferencing program; however, no evaluation records were found of these strategies that justified their interruption.

It is also emphasized that the social appropriation of knowledge should be improved, taking into account the participation of all, including a possible reformulation process, considering their perspectives so that this improvement overcomes potential barriers and recognizes facilitators pointed out as important by the SUS users themselves. In this sense, both initiatives were important strategies: the launch of the guide *Understanding the incorporation of health technologies into SUS: how to get involved* and the event of the same name.

An international survey showed that, among HTA institutions involving society, consultation and information mechanisms are the most common^29^. Since Conitec’s main social participation strategy is PC^23^ and there is an effort to improve participation and transparency, we conclude that the Brazilian institution has strategies aligned with those used by most international HTA agencies.

The obligation to submit all proposals to PC, established with the creation of Conitec, in theory, opens the possibility of (passive) social participation. However, according to Petramale et al.^27^ – technicians and analysts responsible for implementing social participation and HTA processes in the context of the Brazilian Ministry of Health –, this isolated initiative is unlikely to raise social participation.

In 2013, Silva et al.^16^ developed proposals to improve the involvement of the public and patients in HTA processes and incorporation of health technologies in the Brazilian context, from the perspective of SUS. Some of these proposals were implemented by DGITIS, such as: creation of reports in language appropriate to the public; increased public consultation disclosure; use of information and communication technology tools and training for patients.

However, the importance of developing strategies for earlier and more active participation during all stages of the HTA process developed and implemented by Conitec is emphasized. An example of this is surveys, conducted early in the process of drafting the PCDT and which have included the perspective of society since the scope of these documents. However, they deserve to be better disclosed, as some made little or no contribution, as explained in Table 2 .

According to our findings and experience from other agencies^3 , 6 , 29^ the strategies for social participation in Conitec processes that seem to have the greatest implementation potential today are: participation of patient representatives of the issues reviewed at all Conitec plenary sessions; formation of expert patients committees; implementation of active participation methodologies (e.g. citizens’ jury) on the various evaluated topics; holding public hearings; training and support for users of public consultations, as well as improving the methodology to analyze PC’s suggestions.

It is known that a closer relationship with society is possible and we must consider that the spaces of participation are relatively new from an institutional point of view. The results allow us to state that Conitec has advanced in the development and implementation of a variety of social participation strategies in the incorporation of health technologies into SUS, although they need improvement.

For society to be increasingly involved in the health technology incorporation process* in Brazil, it is important that policymakers, managers and their supporters carefully plan and evaluate the strategies to be used. This is one of the weaknesses identified in the Conitec process: the lack of impact evaluation of the strategies and the quality of the suggestions received in PC. It is also important to defend and qualify the established spaces, the actors involved and think about new methodologies, so that many of the challenges are solved or mitigated with practice, especially in the current Brazilian moment, when a recently published decree can bring significant negative impacts to social participation in the country.
