# Supplementary material for: Social participation in the health technology incorporation process into Unified Health System
Source: Rev Saude Publica. 2019 Dec 16;53:109. doi: 10.11606/S1518-8787.2019053001420 (PMC6919280; doi:10.11606/S1518-8787.2019053001420)
Supplement: Translation

# Translation: Participação social no processo de incorporação de tecnologias em saúde no Sistema Único de Saúde

**Authors:** Aline Silveira Silva, Maria Sharmila Alina de Sousa, Emília Vitória da Silva, Dayani Galato

**Affiliations:** I Universidade de Brasília. Faculdade de Ceilândia. Programa de Pós-Graduação em Ciências e Tecnologias da Saúde. Ceilândia, DF, Brasil; II Ministério da Saúde. Secretaria de Ciência, Tecnologia e Insumos Estratégicos – SCTIE. Departamento de Gestão e Incorporação de Tecnologias e Inovação em Saúde – DGITIS. Brasília, DF, Brasil; III Escola Fiocruz de Governo. Brasília, DF, Brasil; IV Universidade de Brasília. Faculdade de Ceilândia. Curso de Farmácia. Grupo de Pesquisa em Acesso aos Medicamentos e Uso Responsável – AMUR. Ceilândia, DF, Brasil

**Keywords:** Avaliação da Tecnologia Biomédica, Participação da Comunidade, Sistema Único de Saúde

## Abstract

**OBJETIVO:**

Descrever o atual processo de participação social na incorporação de tecnologias em saúde no Brasil, no contexto do Sistema Único de Saúde (SUS).

**MÉTODOS:**

Realizou-se um estudo descritivo baseado na análise de registros oficiais das ações da Comissão Nacional de Incorporação de Tecnologias no SUS (Conitec) e do seu sítio eletrônico, desde o início de suas atividades em janeiro de 2012 até dezembro de 2017.

**RESULTADOS:**

Os achados indicam que no Brasil existem instrumentos legais relacionados à participação social na saúde, inclusive no que se refere à avaliação de tecnologias em saúde. No entanto, sua implantação é relativamente recente e tem sido realizada gradualmente. Além dos instrumentos legais (participação de representante do Conselho Nacional de Saúde, realização de consulta pública e previsão de audiência pública), outras estratégias de informação e transparência têm se mostrado aliadas à participação social na incorporação de tecnologias em saúde. Contudo, atividades como audiências públicas, previstas legalmente, ainda não foram realizadas.

**CONCLUSÕES:**

Diversas ações de fomento à participação social foram desenvolvidas ao longo do período analisado, as quais, porém, carecem de avaliação para que ocorra sua manutenção ou aprimoramento. Além disso, percebe-se a necessidade de uma participação social mais qualificada nos vários espaços existentes, incluindo os previstos por lei.

## INTRODUÇÃO

A avaliação de tecnologias em saúde (ATS) é um campo multidisciplinar de análise de políticas públicas, que estuda as implicações clínicas, sociais, éticas e econômicas do desenvolvimento, difusão e uso de tecnologia em saúde, levando-se em consideração aspectos como eficácia, efetividade, segurança, custos, custo-efetividade, entre outros^1 , 2^. Entretanto, os impactos éticos, legais e sociais, direcionados à perspectiva social, são muitas vezes desconsiderados em função dos achados clínicos e econômicos^3^.

Membros do Fórum Global de Políticas da Associação Internacional de ATS ( *Health Technology Assessment International Global Policy Forum)* relataram que há alguns anos os profissionais dessa área focavam na produção de relatórios e processos internos, interagindo de maneira limitada com a sociedade; atualmente, porém, essa perspectiva tem mudado. Os profissionais de ATS têm buscado cada vez mais evidências coloquiais, um termo que abrange diferentes tipos de opinião informal^4^, para complementar os dados de ensaios clínicos randomizados. O objetivo é enfocar vários aspectos, não apenas os clínicos, e, assim, adaptar cada vez mais os processos de ATS aos contextos reais onde essas tecnologias são ou serão utilizadas^5^.

A literatura propõe diferentes termos para se referir aos vários grupos de usuários finais de ATS, como pacientes, consumidores, cidadãos e público. O modelo desenvolvido por Gauvin et al.^6^agrupou esses usuários em duas categorias. O público que constitui a primeira categoria fornece uma perspectiva social ou leiga sobre tecnologias em saúde, que inclui cidadãos, grupos de cidadãos ou representantes eleitos. A segunda categoria compreende aqueles diretamente afetados por uma determinada condição ou tecnologia em saúde e inclui os pacientes, usuários do serviço e entidades que os representam.

O modelo descrito anteriormente enfatiza três principais aspectos: domínios do envolvimento (política organizacional e pesquisa), tipo de público (leigo e diretamente afetados) e nível de envolvimento (informação, consulta e participação)^7^.

Com o crescente engajamento dos pacientes, cada vez mais interados de seus direitos à saúde e participativos no cuidado da própria saúde, há a necessidade de estabelecer meios eficazes para envolver a sociedade civil nos processos de decisão sobre a incorporação de tecnologias. Muitas agências de ATS, em diferentes países, têm considerado diversas maneiras de incorporar as perspectivas dos pacientes em seus modelos e métodos de engajamento social, exigindo assim, uma ATS centrada no paciente^7 , 8^.

Existe falta de consenso terminológico sobre esse envolvimento. Estudos internacionais que abordam o tema utilizam com frequência os termos “envolvimento do público e do paciente”, “envolvimento do público” ou “engajamento de pacientes”^4^. Neste trabalho, adotou-se o termo “participação social”, pois é como a legislação brasileira se refere ao tema, inclusive ao tratar da ATS^9^.

No Brasil, a participação social é uma das diretrizes do Sistema Único de Saúde (SUS) presentes na Constituição Federal^10^e um dos princípios descritos nas Leis 8.080/1990^11^e 8.142/1990^12^. A sociedade construiu o controle social no SUS, com os conselhos e conferências de saúde, definindo espaços de participação social na formulação e deliberação da política pública de saúde^13^. No entanto, essa participação nas sociedades democráticas contemporâneas é fato recente^14^. Embora a participação da comunidade seja assegurada constitucionalmente, os exemplos brasileiros de experiência democrática em saúde nesse âmbito ainda são pontuais^15 , 16^. Além disso, segundo Delduque e Bardal (2008)^15^, a participação social da qual se fala não se restringe ao controle social da saúde. Ou seja, não se refere apenas à formação dos conselhos ou às conferências de saúde, mas sim, a uma participação mais ampla, aquela inerente ao pleno cumprimento da cidadania, permitindo que cidadãos participem verdadeiramente, no sentido habermasiano^17 , 18^da ação comunicativa, dos processos de tomada de decisão em saúde^15^.

A Lei 12.401/2011 define que a incorporação, exclusão ou alteração no SUS de novos medicamentos, produtos e procedimentos, bem como a constituição ou a alteração de protocolos clínicos e diretrizes terapêuticas (PCDT), são atribuições do Ministério da Saúde, assessorado pela Comissão Nacional de Incorporação de Tecnologias no SUS (Conitec)^19^. A estrutura de funcionamento da Conitec é composta por dois fóruns: plenário e secretaria-executiva. Uma das atribuições da secretaria-executiva, exercida pelo Departamento de Gestão e Incorporação de Tecnologias e Inovação em Saúde (DGITIS), é promover ações que favoreçam e estimulem a participação social na incorporação de tecnologias em saúde no SUS^9^; essa atribuição dialoga com a Política Nacional de Participação Social e o Sistema Nacional de Participação Social^20^.

A mesma lei^19^também oficializou a participação social no processo de incorporação de tecnologias, que, anteriormente, não contava com essa previsão legal, tendo havido escassas tentativas de envolvimento do público e do paciente antes da Conitec^16^. Essa participação, portanto, ocorre atualmente por meio do Conselho Nacional de Saúde (CNS), que representa os usuários do SUS como membro do plenário da Conitec; da realização de consulta pública (CP) para todas as recomendações emitidas; e de audiência pública antes da tomada de decisão final, nos casos em que o secretário da Secretaria de Ciência, Tecnologia e Insumos Estratégicos do Ministério da Saúde determinar que a relevância da matéria justifique a sua realização.

Contudo, recentemente, houve a publicação do Decreto 9.759/2019, que “extingue e estabelece diretrizes, regras e limitações para colegiados da administração pública federal”, medida esta que vai na contramão dos avanços sociais, causando insegurança quanto à manutenção da participação social em diversos cenários em nosso país.

Este estudo se propõe a descrever o estado atual da participação social nos processos de gestão de tecnologias em saúde desenvolvidos pela Conitec.

## MÉTODOS

Este trabalho se caracteriza como um estudo de caso sobre a participação social no processo de incorporação de tecnologias desenvolvido pela Conitec no contexto do SUS. Para isso, fizeram-se análise documental^21^e descrição das ações que promoveram a participação social na avaliação e incorporação das tecnologias em saúde, entre janeiro de 2012 e dezembro de 2017. Todas as ações desenvolvidas pela Conitec que tiveram como objetivo alcançar a sociedade e estimular a sua participação foram descritas e seus resultados analisados. Elas foram apresentadas em uma linha do tempo, com a descrição do momento de sua publicação, realização ou implantação, no caso de ações contínuas.

Para a descrição das ações realizadas, foi computado o número de relatórios para a sociedade publicados pela Conitec, o número de consultas públicas realizadas, bem como a contagem e classificação dos autores das contribuições recebidas. A classificação dos autores dessas contribuições foi realizada segundo o modelo de Gauvin et al. (2010)^6^, indicando aqueles diretamente afetados por uma determinada condição ou tecnologia em saúde, e de acordo com o registro do formulário do SUS (FormSUS).

Para obtenção dos dados relacionados aos resultados das ações da Conitec, foram consultadas as planilhas e o sistema de gestão e informações gerenciais do DGITIS, bem como o portal eletrônico da comissão (www.conitec.gov.br).

Este trabalho integra o projeto de pesquisa intitulado “O envolvimento do público e do paciente no processo de incorporação de tecnologias em saúde no Brasil”, que foi aprovado pelo Comitê de Ética da Faculdade de Ceilândia da Universidade de Brasília, sob o parecer 2.225.660.

## RESULTADOS

A Figura 1 apresenta uma linha do tempo com os eventos relacionados à participação social no processo de incorporação de tecnologias em saúde no SUS. As ações estão detalhadamente descritas conforme a seguinte classificação: consulta pública (CP); representação de pacientes no plenário; enquetes; e informação e transparência.

**Figura 1:** Linha do tempo das ações de envolvimento do público e do paciente nos processos de avaliação e incorporação de tecnologias no SUS desenvolvidas desde a criação da Conitec até dezembro de 2017. Fonte: Conitec, 2018.

### Consulta Pública

De acordo com a Política Nacional de Participação Social, consulta pública é um “mecanismo participativo, a se realizar em prazo definido, de caráter consultivo, aberto a qualquer interessado, que visa a receber contribuições por escrito da sociedade civil sobre determinado assunto, na forma definida no seu ato de convocação”^20^.

Na incorporação de tecnologias em saúde no SUS, o objetivo da CP é ampliar a discussão sobre as matérias em análise, perpassando aspectos técnicos, científicos, econômicos e logísticos já identificados. São agregadas também à discussão a visão e as vivências de pacientes, profissionais de saúde, cidadãos e outros atores sociais. Desse modo, após elaborar um relatório técnico, a Conitec o disponibiliza em seu sítio eletrônico para receber contribuições da sociedade por 20 dias. Todas as contribuições são compiladas e levadas ao plenário para análise antes da emissão da recomendação final sobre a incorporação^22^.

Com o intuito de aprimorar as contribuições da CP, em 2014, foi criado um novo formulário para diferenciar os relatos de experiências e perspectivas de pacientes, cuidadores e profissionais de saúde daqueles relatos técnicos e cientiíficos.

Em 2015, iniciou-se a produção de relatórios para a sociedade, que são versões resumidas dos relatórios técnicos da Conitec, disponibilizados junto a estes na CP. São elaborados em linguagem simplificada visando a melhorar a compreensão dos usuários sobre as tecnologias que estão em análise, além de estimular sua participação no processo de ATS. Até dezembro de 2017, foram publicados 76 relatórios para a sociedade.

No período investigado, foram realizadas 257 CP, com 42.630 contribuições, dos diversos atores sociais, principalmente de familiares, amigos ou cuidadores de pacientes; profissionais de saúde; pacientes/usuários do SUS; e atores com interesse no tema ( Figura 2 ). Na análise dos autores das contribuições segundo a classificação de Gauvin et al.^6^, observa-se que quase metade dos participantes (44,6%, n = 19.006) encaixa-se na categoria dos diretamente afetados por uma determinada condição ou tecnologia em saúde (pacientes e usuários do SUS; familiares, amigos ou cuidadores; e grupos, organizações ou associações de pacientes).

**Figura 2:** Tipo de público participante das consultas públicas realizadas pela Comissão Nacional de Incorporação de Tecnologias em Saúde no SUS (conforme autodeclarado nos formulários das consultas públicas), no período de 2012 a 2017. Fonte: Conitec, 2018.

A partir de 2014 ( Figura 1 ), a secretaria-executiva da Conitec objetivou ampliar a informação das CP por meio de algumas mídias sociais digitais, como o *Twitter* institucional, canais de parceiros, *LinkedIn* institucional (que foi criado e encerrado em 2017), sítio eletrônico e listas de *e-mail* . Após a implementação dessas estratégias, a participação social nas CP passou de 2.584 contribuições em 2014 para 13.619 contribuições em 2015 e 16.514 contribuições em 2017 ( Figura 3 ). Além da estratégia de informação adotada, observou-se que temas de grande apelo popular também interferiram no aumento das consultas, como: “Exclusão da betainterferona para o tratamento da esclerose múltipla” (4.846 contribuições) e “Diretrizes de atenção à gestante: a operação cesariana” (3.706 contribuições) em 2015; e “PCDT de profilaxia pré-exposição ao HIV (PrEP)” (3.773 contribuições) e “Fumarato de dimetila para esclerose múltipla” (1.813 contribuições) em 2017.

**Figura 3:** Número de consultas públicas e contribuições recebidas, por ano, até dezembro de 2017. Fonte: Conitec, 2018.

Em alguns casos, as considerações recebidas durante as CP influenciaram a tomada de decisão da comissão, alterando a recomendação inicial de não incorporação para uma recomendação final de incorporação, seja por fornecerem novas evidências científicas e propostas de preço, seja por terem trazido necessidades e preferências dos pacientes que não haviam sido contempladas nos desfechos definidos anteriormente ( Tabela 1 ). No entanto, até o momento, não foram publicadas informações sobre a qualidade ou análise de conteúdo das contribuições, o que poderia orientar a qualificação dessa ferramenta de participação.

**Tabela 1:** Temas que tiveram a recomendação “não incorporação” alterada para “incorporação” após análise de contribuições recebidas nas consultas públicas, até dezembro de 2017.

Ano	Tecnologia	Indicação	Motivo da alteração após CP
2013	Erlotinibe e gefitinibe	Câncer de pulmão de células não pequenas avançado ou metastático mutação EGFR	Apresentação de novas evidências na CP mostrou benefício da tecnologia (acréscimo de sobrevida livre de progressão)
2015	Abatacepte subcutâneo	Artrite reumatoide moderada a grave	Apresentação de nova proposta de preço recebida na CP equiparou a TS analisada ao comparador, em razão da não inferioridade daquela
2015	Rivastigmina adesivo transdérmico	Demência leve e moderadamente grave do tipo Alzheimer	Apresentação de nova proposta de preço recebida na CP equiparou a TS analisada ao comparador, em razão da não inferioridade daquela
2017	Fingolimode	Esclerose múltipla remitente recorrente após falha terapêutica com betainterferona ou glatirâmer	Revisão das linhas de tratamento atualmente preconizadas pelo SUS e disponibilidade de um medicamento genérico
2017	Insulinas análogas de ação rápida	*Diabetes mellitus* tipo I	Reconsideração da importância de outros desfechos, recomendação de incorporação feita mediante negociação de preço com o fabricante
2017	Fumarato de dimetila	Esclerose múltipla remitente recorrente após falha com betainterferona ou glatirâmer	Apresentação de nova proposta de preço recebida na CP equiparou a TS analisada ao comparador, em razão da não inferioridade daquela

### Representação de Pacientes no Plenário da Conitec

O plenário da Conitec possui como um dos membros um representante do CNS. Contudo, a participação de outros representantes da sociedade e de pacientes pode ainda ocorrer de outras formas, as quais, embora escassas, têm importância sob a perspectiva da qualidade das contribuições – evidências coloquiais – para apoiar a tomada de decisão no plenário.

Em alguns momentos, quando solicitado pelo plenário, representantes de pacientes e especialistas no assunto foram convidados para participar das sessões plenárias. Essa participação forneceu à Conitec evidências coloquiais bastante informativas sobre a experiência de pacientes e especialistas no tema^4 , 23 , 24^.

É importante pontuar que, em outros momentos, em reuniões do plenário foi aceita a presença de representantes da sociedade (profissionais ou pacientes) quando solicitada por estes. Nesses casos, os presentes puderam apenas acompanhar a apresentação técnica do tema, mas não a discussão posterior do plenário. Todavia, a legislação prevê a realização de audiência pública quando a relevância da matéria justificar^19^. De acordo com a Política Nacional de Participação Social, a audiência pública é um “mecanismo participativo de caráter presencial, consultivo, aberto a qualquer interessado, com a possibilidade de manifestação oral dos participantes, cujo objetivo é subsidiar decisões governamentais”^20^. No entanto, no período investigado, não foi encontrado registro de utilização desse mecanismo.

### Enquetes

Em 2015, visando a investigar as necessidades e preferências dos usuários do SUS e dar continuidade ao processo de aprimoramento da participação social e transparência dos processos da Conitec, o DGITIS anunciou a realização de enquetes relacionadas aos PCDT. Elas são disponibilizadas no sítio eletrônico da Conitec, normalmente por 20 dias, como consulta prévia, ainda na primeira etapa de construção do PCDT. Assim, a secretaria-executiva pode discutir a proposta inicial do documento e melhorá-la, identificando aspectos que podem não ter sido considerados anteriormente. Essa foi a maneira que a própria comissão encontrou para implementar outras formas de participação social desde o início do processo de elaboração do PCDT, e não apenas na CP.

Dessa forma, quaisquer atores da sociedade podem sugerir informações sobre determinados aspectos das doenças com as quais têm experiência (pessoal ou profissional), melhoras no cuidado de saúde e apelo por novas tecnologias que deveriam ser abordadas. Como essa participação ocorre antes do documento ser elaborado, a sociedade contribui para a construção do escopo do protocolo em questão. Durante o período analisado, foram realizadas 17 enquetes sobre os mais diversos temas ( Tabela 2 ).

**Tabela 2:** Enquetes relacionadas aos protocolos clínicos e diretrizes terapêuticas (PCDT) realizadas pela Conitec, até dezembro de 2017.

N° da Enquete	Ano	Tema	N° de contribuições
01	2015	PCDT de doenças raras	1.140
02	2015	Proposta de elaboração de escopo das diretrizes diagnósticas e terapêuticas para intoxicação por agrotóxicos	38
03	2016	Proposta de atualização dos PCDT publicados em 2012 e 2013	1.054
04	2016	Proposta de elaboração do PCDT destinado ao cuidado do indivíduo com doença de Chagas	37
05	2016	Proposta de escopo do PCDT para leishmaniose visceral e leishmaniose tegumentar	11
06	2016	Proposta de escopo para diretriz brasileira para utilização da angioplastia coronariana com *stent*	10
07	2016	Atualização de PCDT 2014	304
08	2017	Proposta de escopo do PCDT para febre maculosa	5
09	2017	Proposta de escopo do PCDT para malária	1
10	2017	Proposta de escopo do PCDT para chikungunya	4
11	2017	Proposta de escopo de revisão e atualização do PCDT para dependência à nicotina	192
12	2017	Proposta de escopo das diretrizes diagnósticas do mesotelioma maligno de pleura	12
13	2017	Elaboração do guia de submissão de propostas de incorporação de tecnologias pela Conitec	62
14	2017	Proposta de escopo do PCDT hormonioterapia no processo transexualizador	78
15	2017	Proposta de escopo do protocolo de Atenção Básica – dor crônica	116
16	2017	Proposta de escopo da diretriz brasileira de endopróteses em aorta torácica	0
17	2017	Proposta de escopo da diretriz de hipertensão arterial pulmonar	627

### Informação e Transparência

As estratégias adotadas para promover informação e transparência estão apresentadas na Figura 1 .

O sítio eletrônico da Conitec, criado no final do ano de 2014, foi eleito por sua secretaria-executiva como a base das iniciativas de informação e transparência^24^, pois permite acessar todos os relatórios técnicos e as contribuições recebidas nas CP já realizadas, bem como as pautas e atas das reuniões do plenário. De acordo com a análise de acesso institucional, utilizando o *Google Analytics* ®, de janeiro de 2015 a dezembro de 2017 o portal da Conitec teve 482.524 sessões.

Na seção de Participação Social desse sítio eletrônico, criada em junho de 2016, é possível acessar, por exemplo, as enquetes e CP em andamento e encerradas, os relatórios para a sociedade e os *clippings* mensais sobre as atividades da Conitec. De acordo com a análise de acesso institucional feita utilizando o *Google Analytics* ®, essa seção obteve 2.306 visualizações (até dezembro de 2017).

Em 2016, a Conitec lançou o guia *Entendendo a incorporação de tecnologias em saúde no SUS: como se envolver* ^25^direcionado ao público leigo e disponibilizado na seção de Participação Social do sítio eletrônico, a fim de informar e facilitar o engajamento da sociedade. O documento também foi impresso e distribuído em eventos, além de ter sido enviado para diversas instituições.

No segundo semestre de 2017, ocorreu o fórum “Entendendo a incorporação de tecnologias em saúde”. Esse evento foi direcionado a representantes de pacientes, que tiveram a oportunidade de discutir o processo de participação social da Conitec com os membros da secretaria-executiva. Estiveram presentes 103 representantes de pacientes, provenientes de associações dos mais diversos agravos e regiões do país, os quais puderam conhecer melhor os pontos previstos para participação social e fornecer sugestões de melhoria, que foram inclusive apresentadas ao plenário da Conitec posteriormente. Todo o material relativo ao evento foi disponibilizado na seção de Participação Social do sítio eletrônico da Conitec.

Ademais, o DGITIS realizou quinzenalmente, entre agosto de 2016 e dezembro de 2017, videoconferências por meio de um programa denominado “Conitec em evidência”, que teve como finalidade debater e aprofundar temas de relevância da área de gestão e avaliação de tecnologias com os mais diversos atores da área, além de difundir o conteúdo produzido pela Conitec e pelos Núcleos de Avaliação de Tecnologias em Saúde (NATS). Além da participação de várias instituições, via videoconferência, qualquer cidadão pôde acompanhar a transmissão do conteúdo em tempo real e realizar perguntas via *e-mail* e *Twitter* institucional. Apesar de o programa ter sido encerrado em 2017, seu conteúdo permanece disponível no sítio eletrônico da Conitec. No período analisado, foram realizados 25 programas sobre diversas questões relacionadas à ATS, sendo um deles a “Participação social para o fortalecimento da ATS: avanços e desafios”.

## DISCUSSÃO

Existem inúmeros estudos relatando experiências de participação e o aumento da atenção ao envolvimento de pacientes na ATS ao redor do mundo^3 , 5 , 8^.

Os processos de envolvimento diferem entre as agências de ATS no mundo, principalmente no que se refere aos domínios e tipos de envolvimento, aos tipos de público e a quanto esse envolvimento influencia as decisões sobre incorporação de tecnologias em sistemas de saúde^26^.

A experiência brasileira, ainda inicial, mostra ser capaz de se adaptar perante o contexto e as particularidades da ATS nacional. No entanto, apesar dos avanços já obtidos com a oficialização da participação social na ATS^8 , 19 , 22 , 27^, engajar a sociedade nessas atividades continua sendo desafiador.

Além dos mecanismos de participação previstos legalmente, principalmente os relacionados às CP, observamos a experimentação de diversas estratégias pela secretaria-executiva da Conitec desde a sua implementação.

Dado que a representação do CNS enquanto membro da comissão é insuficiente, em razão da impossibilidade de representar o público envolvido em todos os temas avaliados, e ainda que se observem tentativas de representar os pacientes nas sessões plenárias da Conitec quando solicitado pelo plenário, sugerimos que esse mecanismo de participação (ativa) durante as plenárias, com metodologia adequada e previamente definida, seja fortalecido. Outra estratégia a ser explorada seria a audiência pública, um mecanismo previsto^19^, mas ainda não utilizado. Amplamente mencionada na legislação brasileira como um dos principais mecanismos de participação social na gestão pública, tem caráter consultivo e permite que a sociedade expresse seus anseios e opiniões, fornecendo subsídios para a tomada de decisão^28^.

A implementação de estratégias de participação, transparência, tradução e apropriação social do conhecimento desenvolvidas pela Conitec parece estimular o público a participar do processo de incorporação de tecnologias em saúde. Para corroborar essa afirmação, constatou-se um aumento de mais de 400% no número de contribuições anuais nas CP de 2014 para 2015. Pontua-se que algumas ações de comunicação foram descontinuadas no ano de 2017, como o *LinkedIn* e o programa de videoconferências “Conitec em evidência”; porém, não foram encontrados registros de avaliação dessas estratégias que tenham justificado sua interrupção.

Ressalta-se também que a apropriação social do conhecimento deve ser aprimorada, tendo como referência a participação de todos, inclusive em um possível processo de reformulação, considerando suas perspectivas para que esse aprimoramento derrube potenciais barreiras e reconheça facilitadores apontados como de importância pelos próprios usuários do SUS. Nesse sentido, ambas iniciativas constituíram importantes estratégias: o lançamento do guia *Entendendo a incorporação de tecnologias em saúde no SUS: como se envolver* e o evento de mesmo nome.

Um levantamento internacional mostrou que, entre as instituições de ATS que envolvem a sociedade, os mecanismos de consulta e informação são os mais comuns^29^. Uma vez que a principal estratégia de participação social adotada pela Conitec é a CP^23^e que há um esforço em melhorar a participação e transparência, concluímos que a instituição brasileira possui estratégias alinhadas àquelas utilizadas pela maioria das agências de ATS internacionais.

A obrigatoriedade de submeter à CP todas as propostas, estabelecida com a criação da Conitec, abre, em tese, a possibilidade de participação social (passiva). Entretanto, de acordo com Petramale et al.^27^– técnicos e analistas responsáveis pela implementação dos processos de participação social e ATS no contexto do Ministério da Saúde do Brasil –, é pouco provável que essa iniciativa isolada eleve a participação social.

Em 2013, Silva et al.^16^desenvolveram propostas para aprimorar o envolvimento do público e dos pacientes nos processos de ATS e de incorporação de tecnologias em saúde no contexto brasileiro, sob a perspectiva do SUS. Observou-se que algumas dessas propostas foram implantadas pelo DGITIS, como: criação de relatórios em linguagem adequada ao público; ampliação da divulgação das consultas públicas; uso de ferramentas de tecnologia da informação e comunicação e capacitação para pacientes.

No entanto, ressalta-se a essencialidade do desenvolvimento de estratégias para uma participação mais precoce e ativa durante todas as etapas do processo de ATS desenvolvidas e implementadas pela Conitec. Um exemplo disso são as enquetes, realizadas logo no início dos processos de elaboração dos PCDT e que têm incluído a perspectiva da sociedade desde a elaboração do escopo desses documentos. Contudo, merecem ser mais bem divulgadas, visto que algumas tiveram pouca ou nenhuma contribuição, conforme explicitado na Tabela 2 .

De acordo com nossos achados e experiência de outras agências^3 , 6 , 29^, as estratégias para a participação social nos processos da Conitec que parecem ter maior potencial de implementação atualmente são: participação de representantes de pacientes dos temas avaliados em todas as plenárias da Conitec; formação de comitês de pacientes especialistas; implantação de metodologias de participação ativa (por exemplo, júri de cidadãos) sobre os diversos temas avaliados; realização de audiências públicas; treinamento e apoio para os usuários das consultas públicas, além do aprimoramento da metodologia de análise das contribuições.

Sabe-se que é possível uma aproximação cada vez maior com a sociedade e deve-se levar em conta que os espaços de participação são relativamente novos do ponto de vista institucional. Os resultados permitem afirmar que a Conitec avançou no desenvolvimento e na implementação de uma variedade de estratégias de participação social na incorporação de tecnologias em saúde no SUS, embora estas necessitem de aprimoramento.

Para que a sociedade esteja cada vez mais envolvida no processo de incorporação de tecnologias em saúde no Brasil, é importante que formuladores de políticas, gestores e aqueles que os apoiam planejem e avaliem cuidadosamente as estratégias a serem usadas. Esta é uma das fragilidades identificadas no processo da Conitec: a falta de avaliacão do impacto das estratégias e da qualidade das contribuições recebidas nas CP. É importante, ainda, defender e qualificar os espaços estabelecidos, os atores implicados e pensar novas metodologias, a fim de que muitos dos desafios sejam resolvidos ou amenizados com a prática, em especial no momento atual brasileiro, em que um decreto recém-publicado pode trazer impactos negativos de maneira significativa à participação social no país.
